# Interdental Splint—with Case in Practice

**Published:** 1872-05

**Authors:** D. B. M’Lain

**Affiliations:** New Lisbon, Ohio


### Interdental Splint; With Case in Practice.

BY. D. B. M'LAIN, NEW LISBON, OHIO.

In the latter part of March, 1871, Mr. J. M., aged 50, fell from the platform of a railway car in motion, losing his left arm above the elbow, and sustaining very severe injuries of the head and face, the bones of the face between the eyes and mouth having eight distinct fractures. The nose, malar bones and superior maxilla were found to be perfectly loose and independent of each other. The upper edge of the right malar bone was fractured near its articulation with the superior maxilla, and the articulation of the maxilla and the nasal bone was broken. This caused all the deformity that resulted in the face, and that is slight.

These injuries, as well as a transverse fracture in each ramus of the inferior maxilla, were skillfully treated by a surgeon before I saw the case. But the surgeon from some cause almost entirely neglected an oblique fracture of the inferior maxilla, starting between the left central and lateral incisors and slanting toward the ramus at an angle of about forty-five degrees. All the surgeon had done was to twist a silver wire around the two teeth adjoining the fracture, and as they were very loose the wire of course came off, and he made no further effort.

Six weeks after the accident the patient was brought home, and his home surgeon called me in to file a notch in the tooth to retain a wire, which, when I saw the case, I refused to do. I said that I could make a splint and adjust it, when he at once told me to take the case as far as the mouth was conerned.

Bean's method I consider too complicated for a dentist in ordinary practice to undertake, and in this case impracticable.

On examination I found that it was a simple fracture, and of such a nature that by grasping the jaw on each side and bringing it accurately together it was very firm, the other fractures having firmly united. I warmed a piece of parraffine and wax, about as long as my thumb and twice as thick, having it very soft.

Having the head firmly held by an assistant, I introduced the wax, and grasping the jaw and getting it accurately joined, I directed him to bite very gently.

I simply took an antagonizing impression just as I would for a partial denture. Before the accident his superior and inferior incisors were nearly a half an inch apart when the molars occluded.

I let him bite until the molars almost touched. I took the wax from his mouth and trimmed it, leaving the lower surface intact, so that it would rest on and embrace the points of all the inferior teeth as far back as the second bicuspids, and the superior teeth from the cuspid to the second bicuspids on each side, leaving an opening under the superior incisors about an inch long and half an inch wide for him to take liquid nourishment, which he could do, as well as talk, with the greatest ease and comfort after the splint was finished and applied. The wax I reproduced in whalebone rubber, polished and placed it in the mouth, having passed a silver wire around the cuspids (instead of the loose incisors) drawing the parts firmly together. When all was in place and the jaws drawn firmly into the splint by a modification of the four-tailed bandage, it was as secure and comfortable as one could wish. The splint was retained only five days, and then taken off without my knowledge, but the uniting process had begun, and as he was exceedingly careful, even to sleeping in his easy chair for several weeks, no harm was done.

The surgeon who first treated the case, not being aware of the peculiar antagonism of his teeth, tried, while the superior maxilla was broken up and free, to get the jaws and teeth into a natural position before the patient knew what he was trying to do.

The result was that the entire lower part of the superior maxilla is set almost the width of the molars to the right, making mastication a little difficult. When I took the case his gums had become spongy from using liquid food, and had a hypertrophied appearance. The upper teeth, naturally very short, were almost hidden. I ordered friction with a soft brush three times a day, using a carbolic acid wash. The case is now all right except the slight deformity before mentioned.

The gentleman is one of our prominent lawyers, and in practice.

